# Neoadjuvant Chemotherapy With Gemcitabine/Cisplatin/S‐1 for Resectable Biliary Tract Cancer With FDG‐PET‐Positive Lymph Node Metastasis (KHBO1201): A Multicenter Phase II Trial

**DOI:** 10.1002/jhbp.70030

**Published:** 2025-11-16

**Authors:** Satoshi Ogiso, Etsuro Hatano, Satoru Seo, Masashi Kanai, Shogo Kobayashi, Hiroshi Wada, Hidetoshi Eguchi, Tatsuya Higashi, Hiroaki Nagano, Tatsuya Ioka

**Affiliations:** ^1^ Department of Surgery, Graduate School of Medicine Kyoto University Kyoto Japan; ^2^ Department of Surgery Kochi Medical School Nankoku‐City Kochi Japan; ^3^ Department of Clinical Oncology Kyoto University Hospital Kyoto Japan; ^4^ Cancer Treatment Center Kansai Medical University Hospital Osaka Japan; ^5^ Department of Gastroenterological Surgery, Graduate School of Medicine Osaka University Osaka Japan; ^6^ Department of Gastroenterological Surgery Osaka International Cancer Institute Osaka Japan; ^7^ Department of Molecular Imaging and Theranostics Institute for Quantum Medical Science, National Institutes for Quantum Science and Technology Chiba Japan; ^8^ Department of Gastroenterological, Breast and Endocrine Surgery, Graduate School of Medicine Yamaguchi University Yamaguchi Japan; ^9^ Department of Oncology Center Yamaguchi University Hospital Yamaguchi Japan

**Keywords:** biliary tract neoplasms, chemotherapy, lymphatic metastasis, neoadjuvant therapy, positron‐emission tomography

## Abstract

**Purpose:**

To evaluate the safety and efficacy of neoadjuvant gemcitabine, cisplatin, and S‐1 (GCS) chemotherapy for resectable biliary tract cancer (BTC) and FDG‐PET‐positive lymph nodes in a multicenter phase II study (KHBO1201).

**Methods:**

Patients with resectable BTC (intrahepatic/extrahepatic bile duct, gallbladder, or ampullary cancers) and FDG‐PET‐positive lymph nodes received GCS chemotherapy: gemcitabine/cisplatin on Day 1 and oral S‐1 for 7 days, repeated every 2 weeks for 3–6 cycles. Surgery was planned 4–8 weeks later if the tumor was deemed resectable. The primary endpoint was the curative resection rate. Secondary endpoints were the completion rate, radiological response, radiological/pathological complete response (CR) of FDG‐PET‐positive lymph nodes, and 1‐year survival (UMIN000009831).

**Results:**

Twenty‐five patients were enrolled. Twenty‐three (92%) completed GCS without treatment‐related deaths; grade 3 biliary infection occurred in 8.0%. Curative resection was achieved in 60% with a morbidity rate of 40%. The radiological response rate was 13%, and the radiological and pathological CR rates of FDG‐PET‐positive lymph nodes were 24% and 28%, respectively. The 1‐year survival rate was 75%.

**Conclusions:**

Neoadjuvant GCS chemotherapy is safe, feasible, and potentially effective for resectable BTC with FDG‐PET‐positive lymph nodes. A randomized phase III trial (JCOG1920) is underway to compare neoadjuvant GCS chemotherapy with upfront surgery (jRCTs031200388).

## Graphical abstract

Ogiso and colleagues conducted a multicenter phase II trial evaluating neoadjuvant gemcitabine, cisplatin, and S‐1 for resectable biliary tract cancer with FDG‐PET‐positive lymph node metastasis. The regimen was safe and feasible, achieved a 60% curative resection rate with acceptable toxicity, and showed encouraging survival, supporting further evaluation in randomized phase III trials.

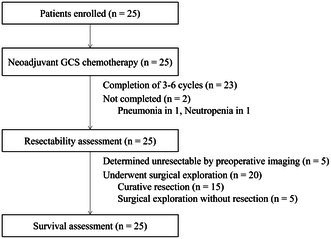

**Abbreviations**
ALT: alanine aminotransferase
AST: aspartate aminotransferase
BSA: body surface area
BTC: biliary tract cancer
CR: complete response
CTCAE: common terminology criteria for adverse events
ECOG: Eastern Cooperative Oncology Group
FDG‐PET: fluorodeoxyglucose positron emission tomography
GC: gemcitabine and cisplatin
GCS: gemcitabine, cisplatin, and S‐1
G‐CSF: granulocyte colony stimulating factor
GS: gemcitabine and S‐1
HR: hazard ratio
JCOG: Japan Clinical Oncology Group
KHBO: Kansai Hepatobiliary Oncology Group
MST: median survival time
NCCN: National Comprehensive Cancer Network
OS: overall survival
PD: progressive disease
PFS: progression‐free survival
PR: partial response
RECIST: response evaluation criteria in solid tumors
RFS: relapse‐free survival
SD: stable disease
SUV: standardized uptake value
UMIN: University Medical Information Network

## 1 Introduction

Surgery remains the only potentially curative treatment for biliary tract cancers (BTC), including intrahepatic and extrahepatic (perihilar and distal) BTC, gallbladder cancer, and ampullary cancer. However, 49.6% of BTC cases are far advanced to be resected [1] and, even when surgery is achieved, the surgical cure rate is very low, with 5‐year overall survival rates (OS) of less than 50% and a relapse rate of more than 60% [1]. Lymph node metastasis is a dismal prognostic factor in resected BTC [1, 2, 3, 4, 5]. In particular, patients with FDG‐PET‐positive lymph node metastasis had poor survival (91 days of mean DFS after resection of intrahepatic BTC [6] and 5.6 months of median OS after resection of extrahepatic BTC [7]).

Chemotherapy for BTC has been developed primarily for patients with unresectable diseases. The first prospective multicenter phase III study in this field, the ABC‐02 study, compared the combination of gemcitabine and cisplatin (GC) with gemcitabine monotherapy and found that the combination regimen significantly prolonged MST (from 8.1 to 11.7 months; *p* < 0.001) [8]. A randomized phase III clinical trial demonstrated that the combination of gemcitabine plus S‐1 (GS) was non‐inferior to gemcitabine plus cisplatin for advanced or recurrent BTC [9]. Furthermore, we reported the safety and efficacy of adding S‐1 to gemcitabine/cisplatin (GCS) combination regimen for advanced (unresectable or recurrent) BTC [10, 11]. After determining the recommended dose in a phase I trial [10, 11], a phase II trial evaluated the efficacy in 50 patients with advanced BTC and showed promising results with an MST of 16.2 months and a 1‐year survival rate of 60% [10, 11]. This led us to conduct a randomized phase III study of GCS versus GC chemotherapy for unresectable BTC, which recently demonstrated that GCS was superior to GC in terms of response rate and OS [12], and has been followed by increasing use of GCS in real‐world practice in Japan [13].

In contrast to the advances in treatments for unresectable or recurrent BTC, only a few studies have assessed the effectiveness of neoadjuvant therapy for resectable BTC. Neoadjuvant chemotherapy offers several theoretical advantages over upfront surgery, including early delivery of systemic therapy for almost all patients, the potential for better tolerance of multi‐agent regimens, and the probability of controlling micrometastasis, which may ultimately improve OS. This study aimed to evaluate the efficacy and safety of neoadjuvant chemotherapy using the GCS regimen for resectable BTC with FDG‐PET‐positive lymph node metastasis.

## 2 Patients and Methods

### 2.1 Study Design

**FIGURE 1:**
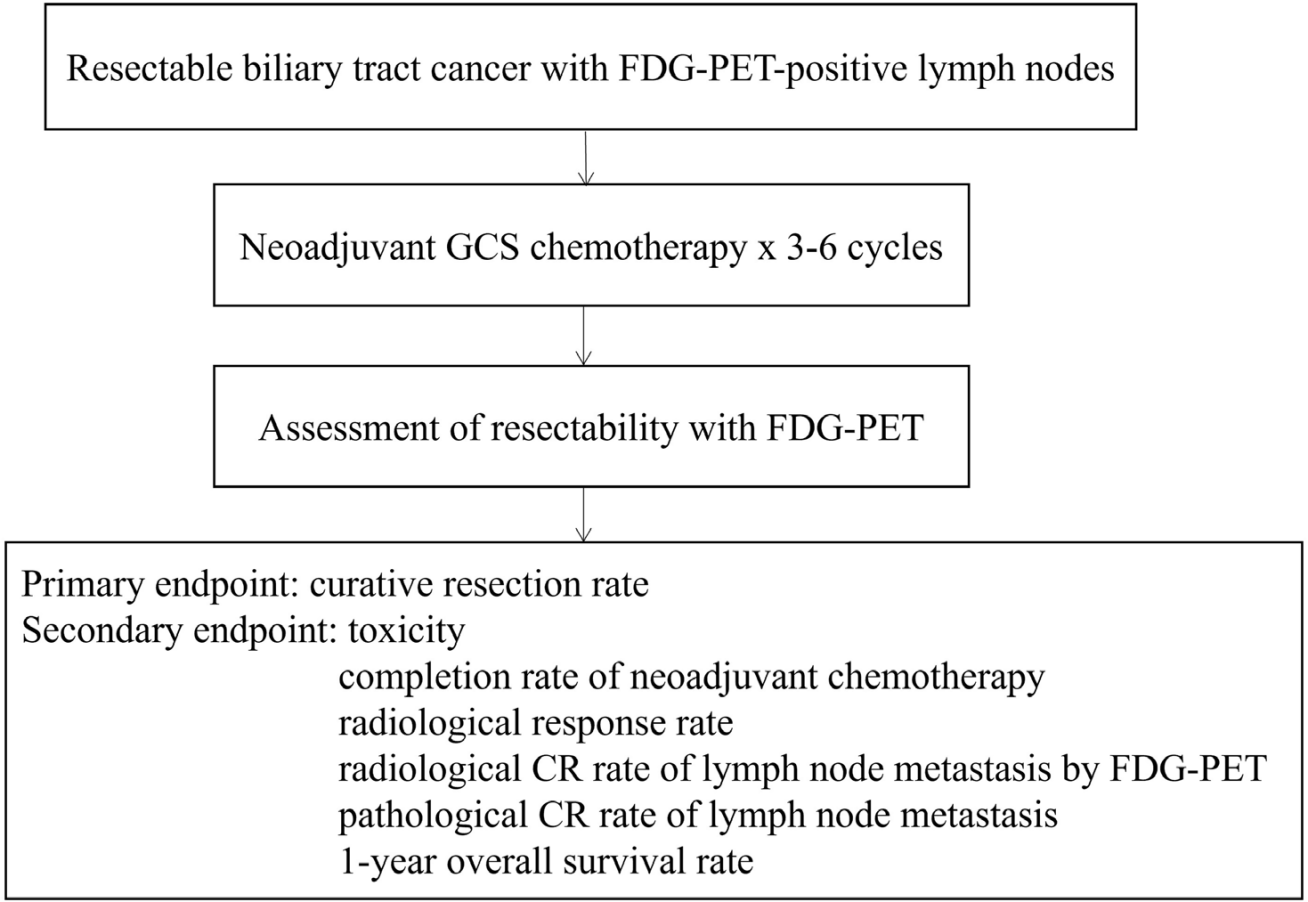
The study flow chart. GSC, gemcitabine, cisplatin, and S‐1; LN, lymph node.

This multicenter, single‐arm, phase II study (KHBO1201) was conducted by the Kansai Hepatobiliary Oncology Group (KHBO) at three academic institutions. The flowchart of the study is presented in Figure 1. The primary objective of this study was to evaluate the efficacy and safety of preoperative chemotherapy with Gemcitabine/Cisplatin/S‐1 in BTC with lymph node metastasis on FDG‐PET.

### 2.2 Inclusion Criteria

This study included patients who met the following criteria: resectable BTC with FDG‐PET positive lymph node; presence of histologically or cytologically confirmed adenocarcinoma of the biliary tract, or radiologically diagnosed biliary tract cancer when pathological confirmation was not feasible (intra‐ or extra‐hepatic cholangiocarcinoma, gallbladder cancer, or ampulla of Vater cancer); Eastern Cooperative Oncology Group performance status of 0–1; age ≥ 20 years; no prior chemotherapy or radiotherapy except for adjuvant chemotherapy, which had been completed at least 6 months before enrolment; adequate bone marrow (neutrophil count ≥ 1500/mm, platelet count ≥ 100 000/mm), liver [total bilirubin ≤ 3.0 mg/dL, aspartate aminotransferase (AST) ≤ 150 IU/L, alanine aminotransferase (ALT) ≤ 150 IU/L], and renal functions (calculated creatinine clearance using Cockcroft and Gault formula ≥ 60 mL/min); and adequate oral intake. Regional lymph node metastases (hilar, periduodenal, and peripancreatic) were considered resectable at baseline, based on preoperative imaging. In selected cases, para‐aortic or pericaval lymph node metastases were eligible only if the tumor burden was limited and curative resection was considered achievable in cases where FDG uptake disappeared following chemotherapy. All cases were reviewed by a multidisciplinary tumor board at each participating institution. The exclusion criteria were as follows: pulmonary fibrosis or interstitial pneumonia, severe heart disease, uncontrollable diabetes mellitus, active infection, pregnancy or lactation, age within the childbearing range for women (unless effective contraception was being used), severe drug hypersensitivity, mental disorder, watery diarrhea, moderate or marked pleural effusion or ascites necessitating drainage, and other serious medical conditions.

### 2.3 The Definition of FDG‐PET‐Positive Lymph Node

**FIGURE 2:**
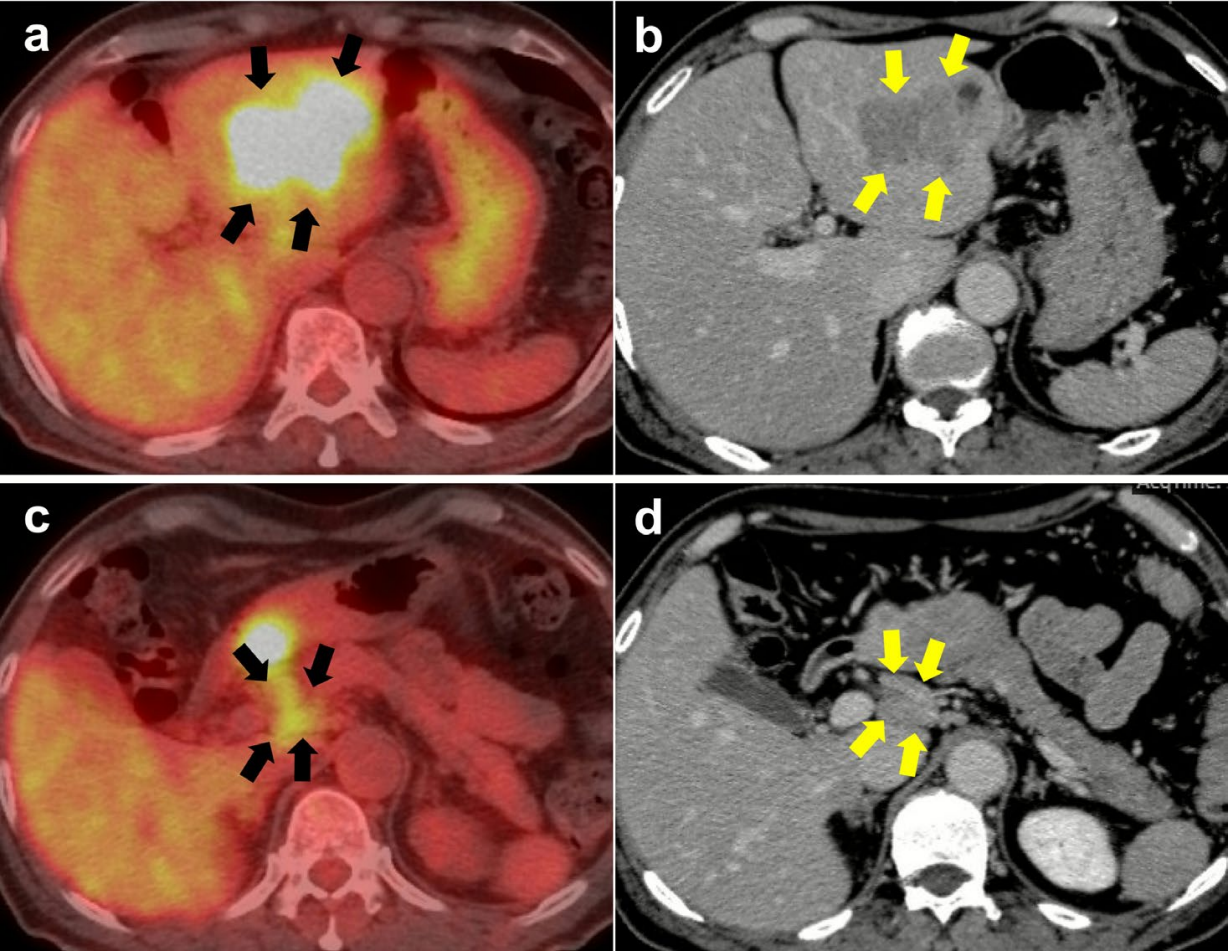
Representative case of a patient with FDG‐PET‐positive lymph node included in the study. (a) FDG‐PET CT and (b) contrast‐enhanced CT showing a tumor in the left liver and (c) FDG‐PET CT and (d) contrast‐enhanced CT showing an enlarged FDG‐avid lymph node in the peripancreatic region.

FDG‐PET scans were performed at baseline (just before study enrollment) and repeated within 4 weeks after completing the neoadjuvant GCS chemotherapy. An FDG‐PET‐positive lymph node was defined as a lymph node meeting all the following criteria: (1) location in the perihilar, retroduodenal, or paraaortic region; (2) a minimum short‐axis diameter of 10 mm or more on CT images; and (3) presence of a greater FDG uptake compared with surrounding non‐tumorous tissue (Figure 2) such as the erector spinae iliocostalis/longissimus/multifidus muscles. Paraaortic lymph node metastasis was considered distant disease regardless of the tumor location.

### 2.4 Chemotherapy

Gemcitabine/cisplatin was infused at a dose of 1000/25 mg/m^2^ over 30/60 min on Day 1. S‐1 was administered orally twice daily for seven consecutive days. Doses of S‐1 were calculated according to body surface area (BSA) as follows: BSA < 1.25 m^2^, 80 mg/day; 1.25 m^2^ ≤ BSA < 1.5 m^2^, 100 mg/day; and BSA ≥ 1.5 m^2^, 120 mg/day. The protocol treatment was continued every 2 weeks for three cycles, with an additional three cycles administered as needed. Granulocyte colony‐stimulating factor (G‐CSF) was administered in cases of neutrophil count < 500/mm^3^, febrile Grade 3 neutropenia (< 1000/mm^3^), or as secondary prophylaxis following such events. Other standard institutional supportive measures such as antibiotics were allowed at the discretion of the treating physician. The details of chemotherapy have been previously described [12].

### 2.5 Surgery

Resectability was evaluated 2–4 weeks after the last administration of neoadjuvant GCS chemotherapy. Surgery was performed 4–8 weeks after the last administration of neoadjuvant GCS chemotherapy only if the tumor was regarded as resectable during the preoperative workup.

### 2.6 Ethics Statement

The study protocol was approved by the Protocol Review Board at each participating institution (representative IRB approval number: C0684, Kyoto University). Written informed consent was obtained from all patients. Patient registration and data management were conducted at a data center of the KHBO. This trial was registered as UMIN000009831.

### 2.7 Statistical Analysis

The primary endpoint was curative resection rate. The secondary endpoints were safety, response rate, completion rate of neoadjuvant GCS chemotherapy, radiological response rate which was assessed according to the response evaluation criteria in solid tumors (RECIST) version 1.1, radiological and pathological complete response (CR) rates in FDG‐PET‐positive lymph nodes, and one‐year survival rate. Pathological CR was defined as the absence of viable tumor cells in the lymph nodes that had been FDG‐PET‐positive at baseline. Although lymph node metastasis was not histologically confirmed prior to GCS chemotherapy, this definition was based on the high positive predictive value of FDG‐PET combined with CT (≥ 10 mm short axis) as previously reported [6, 7, 14, 15]. Adverse events were defined according to the Common Terminology Criteria for Adverse Events (CTCAE) version 4.0. A threshold and an expected rate of curative resection were assumed as 50% and 75%, respectively, based on our previous report of a curative resection rate of approximately 60% [7]. The minimum sample size was calculated as 23 patients, with a one‐sided significance level of 0.9 and a power of 80%. After considering the potential patient dropouts, a sample size of 25 patients was determined. The SUV change ratio was defined as the SUV after GCS chemotherapy divided by the SUV before GCS chemotherapy. OS was defined as the time from the date of registration to death from any cause. Progression‐free survival (PFS) was defined as the time from the date of registration to disease progression or death from any cause, whichever occurred first. Survival rates were analyzed using the Kaplan–Meier method. The JMP software program (SAS Institute, Cary, NC, USA) was used for all the statistical analyses.

## 3 Results

**FIGURE 3:**
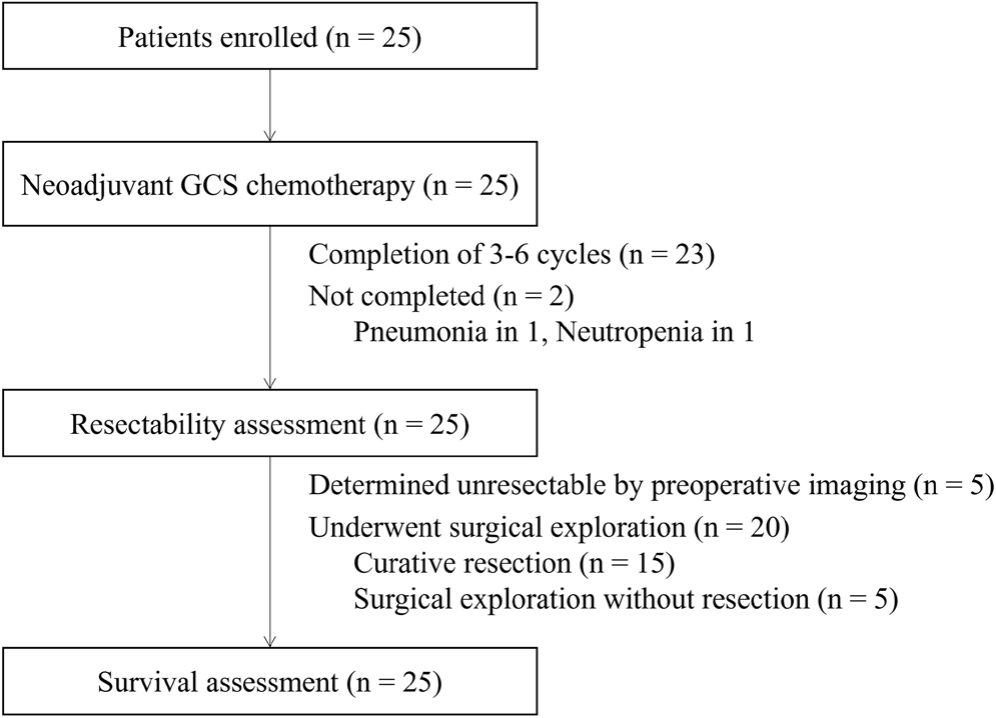
Overview of the study's outcomes. GSC, gemcitabine, cisplatin, and S‐1; CR, complete response.

Between July 2013 and September 2016, 25 BTC patients were enrolled in this study. Background characteristics and clinicopathological data, together with treatment outcomes, are summarized in Table 1 and Figure 3. There were 10 male and 15 female patients with a median age of 65 years. The most common tumor location was intrahepatic in 12 of 25 patients (48%) and the second most common was gall bladder in 6 (24%). Metastatic disease was observed in one patient with paraaortic positive lymph nodes (4%). According to UICC stage classification, stage IVA was the most common (68%). The SUV was higher in the main tumor than in the FDG‐PET‐positive lymph node (6.7 vs. 3.3, *p* < 0.01).

**TABLE 1:** Background characteristics, clinicopathological findings, and treatment outcomes.

Male/Female, *n* (%)	10 (40)/15 (60)
Age, median years (range)	65 (37–82)
PS 0/1, *n* (%)	24 (96)/1 (4)
Tumor location, *n* (%)	
Intrahepatic	14 (56)
Perihilar	4 (16)
Extrahepatic	9 (36)
Gall bladder	6 (24)
Ampullary	1 (4)
T stage (1/2/3/4), *n* (%)	0 (0)/4 (16)/11 (44)/10 (40)
Metastatic disease (0/1), *n* (%)	24 (96)/1 (4)
UICC stage (I/II/III/IVA/IVB), *n* (%)	0 (0)/5 (20)/2 (8)/17 (68)/1 (4)
Serum tumor marker levels before NAC GCS	
CA 19–9, median (range), U/mL	204 (2–4313)
CEA, median (range), ng/mL	3 (0–18)
SUV	
Main tumor, median (range)	6.7 (3.6–14.1)
FDG‐PET‐positive LN, median (range)	3.3 (1.3–20.6)
Completion of NAC GCSx3 (Y/N), *n* (%)	23 (92)/2 (8)
Response rate (CR/PR/SD/PD), *n* (%)	0 (0)/3 (13)/18 (75)/3 (13)
Radiological CR of FDG‐PET‐positive LN (Y/N), *n* (%)	5 (24)/16 (76)
Serum tumor marker levels after NAC GCS	
CA 19–9, median (range), U/mL	110 (2–3220)
CEA, median (range), ng/mL	3 (0–37)
SUV after NAC GCS	
Main tumor, median (range)	4.5 (3.0–9.5)
FDG‐PET‐positive LN, median (range)	3.1 (1.9–10.2)
Curative R0 resection, *n* (%)	15 (60)
Major hepatectomy	10 (67)
PVE before major hepatectomy	5 (50)
Combined with extrahepatic bile duct resection	5 (50)
Combined with vascular resection	2 (20)
Minor liver resection	1 (7)
Extrahepatic bile duct resection	1 (7)
Pancreatoduodenectomy	3 (20)
Major complication ≥ CD grade III	6 (40)
Postoperative chemotherapy after curative resection	6 (40)
Pathological CR of FDG‐PET‐positive LN (Y/N), *n* (%)	5 (28)/13 (72)

*Note:* The data at the time of enrollment and after neoadjuvant GCS chemotherapy is presented for the 25 enrolled patients. The data at the time of surgery and pathology is presented for the 15 patients who underwent curative resection.

Abbreviations: CA19‐9, carbohydrate antigen 19‐9; CD, Clavien‐Dindo classification; CEA, carcinoembryonic antigen; CR, complete response; GCS, gemcitabine, carboplatin, and S‐1 chemotherapy; LN, lymph node; NAC, neoadjuvant chemotherapy; PD, progressive disease; PR, partial response; PS, performance status; PVE, portal vein embolization; SD, stable disease; SUV, standardized uptake value; UICC, Union for International Cancer Control.

A total of 3–6 cycles of GCS chemotherapy were completed in 23 patients (3 cycles in 21 and 6 cycles in 2), without any dose reduction. In the two patients who received six cycles, one continued chemotherapy due to impaired liver function despite disease control, while the other received additional cycles because para‐aortic lymph node metastasis was present at baseline, and a deeper response was desired prior to surgery. Chemotherapy was discontinued in two patients because of Grade 3 pneumonia and Grade 4 neutropenia; these two patients did not undergo follow‐up FDG‐PET scans, as they were among the four patients who did not undergo follow‐up scans. As for biliary infection, Grade 3 events were observed in 8.0%. Table 2 summarizes adverse events associated with neoadjuvant GCS chemotherapy, based on the highest grade experienced by each patient during the treatment course.

**TABLE 2:** Adverse events associated with neoadjuvant GCS chemotherapy.

	Any grade	Grade 3–4
Leukopenia	60.0%	28.0%
Neutropenia	72.0%	40.0%
Febrile neutropenia	4.0%	4.0%
Anemia	84.0%	4.0%
Thrombocytepenia	60.0%	4.0%
Creatinine increase	20.0%	0.0%
AST increase	64.0%	8.0%
ALT increase	56.0%	8.0%
Alb decrease	60.0%	0.0%
T‐Bil increase	44.0%	0.0%
Appetite loss/nausea	68.0%	0.0%
Diarrhea	4.0%	0.0%
Fatigue	68.0%	0.0%
Stomatitis	8.0%	0.0%
Rash	8.0%	0.0%
Biliary infection	8.0%	8.0%
Pharyngitis	4.0%	0.0%
Lower leg edema	4.0%	0.0%
Fever	8.0%	0.0%
Constipation	32.0%	0.0%
Pneumonia	4.0%	4.0%

*Note:* Toxicity rates are presented based on the highest grade experienced by each patient during the neoadjuvant GCS chemotherapy.

Abbreviations: ALB, albumin; ALT, alanine aminotransferase; AST, aspartate aminotransferase; T‐Bil, total bilirubin.

Radiological response rate (CR+ partial response [PR]) was 13%, while CR was 0%, stable disease (SD) was 75% and progressive disease (PD) was 13%. As for FDG‐PET‐positive lymph nodes, FDG uptake disappeared in 5 of 21 patients (24%). The SUV in the main tumor (median, 4.5 vs. 6.7, *p* = 0.07) and FDG‐PET‐positive lymph nodes (median, 3.1 vs. 3.3, *p* = 0.09) tended to decrease after GCS chemotherapy compared to before the treatment, although the difference was not statistically significant. SUV change ratio was a median of 0.77 (0.33–1.64) for the main tumor and 0.88 (0.46–1.82) for the lymph node, respectively, with a significantly greater decrease in SUV change ratio of the main tumor (*p* = 0.04).

After GCS chemotherapy, BTC was deemed resectable in 20 patients and unresectable in five. Among these 5 patients, three were oncologically unresectable due to PD, one was medically unfit after severe pneumonia, and one chose against surgery at his own discretion, despite initially enrolling with surgical intent. The patient who developed grade 4 neutropenia received only two cycles of GCS chemotherapy, remained in SD without a follow‐up FDG‐PET scan, and subsequently underwent curative resection.

The 20 patients (80%) underwent surgery, with a median interval of 44 days after the completion of GCS chemotherapy, while the interval to surgery extended beyond 10 weeks in 3 of these patients due to waiting for liver hypertrophy after percutaneous transhepatic portal embolization (PTPE) or recovery from liver dysfunction. Of these, 15 patients underwent curative R0 resection (60%), and the types of resections are listed in Table 1. Major hepatectomy, defined as removal of ≥ 3 segments, was performed in 10 of 15 patients (67%), after portal vein embolization in 5, and combined with extrahepatic resection in 5 and vascular resection in 2. Minor hepatectomy was performed in 1 (extended right posterior sectionectomy) and pancreatoduodenectomy in 3. Of these, major complications were observed in 6 (40%), including 3 biliary leakages at bilioenteric anastomosis, one ascites, one wound abscess, and one death secondary to postoperative liver failure. The remaining five patients did not undergo complete resection due to peritoneal dissemination in 3 patients, severe fatty liver in 1 patient, and para‐aortic lymph node metastases in 1 patient. Among the lymph nodes that were FDG‐PET positive before chemotherapy, pathological CR was observed in 5 of 18 patients (28%) and associated with lower SUV in the main tumor before GCS chemotherapy (5.3 vs. 13.3, *p* = 0.03). The SUV change ratio of the main tumor (0.65 vs. 0.94, *p* = 0.14) or FDG‐PET‐positive lymph node (0.83 vs. 1.09, *p* = 0.12) did not correlate with the pathological CR of FDG‐PET‐positive lymph nodes. As for the performance of post‐chemotherapy FDG‐PET in detecting persistent lymph node metastasis, the sensitivity was 82%, the specificity was 60%, the positive predictive value was 82%, and the negative predictive value was 60%. In one case of para‐aortic lymph node metastasis confirmed intraoperatively, FDG uptake in the PET‐positive lymph nodes disappeared on FDG‐PET after GCS chemotherapy; however, residual cancer was histologically confirmed, and para‐aortic lymph node metastasis was observed during surgery.

**FIGURE 4:**
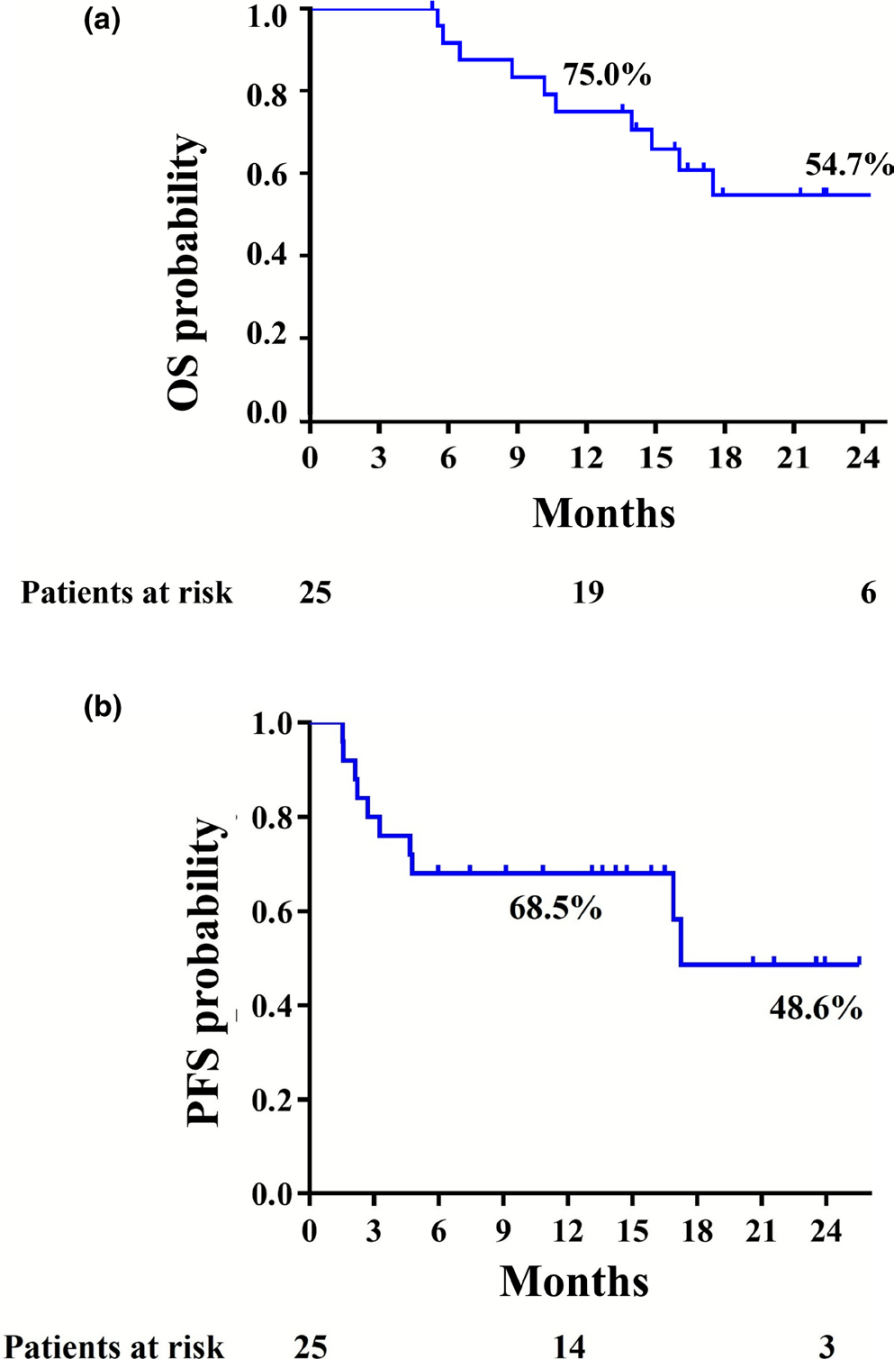
(a) Overall and (b) progression‐free survival curves of the 25 patients included in the study. OS, overall survival; PFS, progression‐free survival.

In the OS analysis, during a median follow‐up of 16.1 (5.2–30.3) months, 11 events (deaths) occurred, and 14 patients were censored at the time of last follow‐up. The 1‐ and 2‐year OS was 75.0% and 54.7%, respectively (Figure 4a), and the median OS has not been reached. In the PFS analysis, during a median follow‐up of 13.1 (1.5–25.5) months, 15 events (5 recurrences and 10 disease progressions) occurred, and 10 patients were censored at the time of last follow‐up. The median PFS was 17.3 months with 1‐ and 2‐year PFS rates of 68.5% and 48.6% (Figure 4b). Among the 15 patients who underwent curative resection, the 1‐ and 2‐year OS rates were 86.7% and 79.4%, respectively, while the 1‐ and 2‐year RFS rates were 73.3% and 61.1%, respectively.

## 4 Discussion

Patients with BTC and lymph node metastasis have poor survival outcomes even after macroscopically curative resection (R0 and R1) [1] and potent adjuvant chemotherapy [16]. However, an effective neoadjuvant therapy for resectable BTC has not been fully investigated or established. This phase II study demonstrated that neoadjuvant GCS chemotherapy is safe and feasible for patients with resectable BTC and FDG‐PET‐positive lymph nodes, with limited toxicity, high tolerance, and a completion rate of 92%. Additionally, it achieved a curative resection rate of 60%, although this did not reach the expected rate, along with radiological and pathological CR rates of 24% and 28%, respectively, for FDG‐PET‐positive lymph nodes. While preliminary survival data were encouraging (1‐year OS of 75.0% and 1‐year PFS of 68.5%), the study was not powered to conclusively assess efficacy. These observations suggest potential benefits of neoadjuvant GCS chemotherapy, which warrant further investigation in larger trials.

Adjuvant chemotherapy is expected to improve the survival probability in patients with resected BTC and has been actively investigated to date. The BILCAP study demonstrated superior mean RFS of adjuvant chemotherapy with oral capecitabine over surgery alone (24.4 vs. 17.5 months, HR 0.75, *p* = 0.033) [17], and the ASCOT study demonstrated superior 3‐year OS rate of adjuvant S‐1 over surgery alone (77.1 vs. 67.6%, one‐sided *p* = 0.0080) [16]. However, survival outcomes in patients with positive lymph nodes remained very low despite such adjuvant chemotherapy. The 5‐year OS was 35.2%–50.0% in these patients, compared to 61.7%–72.7% in patients without lymph node metastasis [16, 17]. Similarly, the 5‐year RFS was 32.6% with positive lymph nodes, versus 62.9% without lymph node metastasis [16]. Therefore, the development of efficient neoadjuvant therapy is warranted to further improve survival outcomes in resectable BTC with lymph node metastasis [18].

A phase III randomized controlled trial, the GAIN trial, presented at the 2025 ASCO Annual Meeting [19], represents an important milestone in the neoadjuvant treatment of resectable BTC, irrespective of lymph node status. The study demonstrated improved OS with GC chemotherapy compared to upfront surgery (median OS, 27.8 vs. 14.6 months; HR 0.46 [95% CI, 0.22–0.96]; *p* = 0.04), as well as a higher R0 resection rate (62.5% vs. 33.3%). Nonetheless, the suboptimal outcomes highlight the need to further refine neoadjuvant strategies, potentially by incorporating a more intensive triplet regimen. In this context, the present phase II study adopted GCS chemotherapy as the neoadjuvant regimen and achieved a 60% curative resection rate comparable to that reported in the GAIN trial, despite targeting a more aggressive subset. Although the curative resection rate did not reach the expected 75%, encouraging secondary outcomes support further evaluation in the ongoing randomized phase III trial (JCOG1920) (jRCTs031200388) [20], which will clarify potential survival benefits as well as any impact on resection rates.

This study demonstrated the safety and feasibility of neoadjuvant GCS chemotherapy, with a high completion rate of 92% and acceptable toxicity profiles. One concern with potent neoadjuvant chemotherapy is the risk of chemotherapy‐associated complications, especially cholangitis. In this study, the incidence of Grade 3 biliary infection was 8.0%, with no Grade 4 biliary infections, and no surgeries were postponed or canceled due to biliary infection. These results suggest that neoadjuvant GCS chemotherapy can be safely administered without increasing perioperative risks. Close monitoring of hematological parameters and signs of infections, appropriate dose adjustments, and early treatment are warranted to minimize the risk of treatment‐related complications and maximize the chance of curative resection. In contrast to the high completion rate of the neoadjuvant GCS chemotherapy revealed by this study, previous phase III studies on adjuvant chemotherapy for BTC have reported low completion rates of 49%–72% with 77%–96% relative dose intensity [16, 17, 21, 22], due to the high invasiveness of BTC surgery [23, 24, 25, 26, 27] and increased toxicity of adjuvant chemotherapy, especially after major hepatectomy [28, 29]. This higher tolerability of neoadjuvant chemotherapy compared to adjuvant chemotherapy is another advantage of preoperative neoadjuvant chemotherapy. Another concern in the use of neoadjuvant chemotherapy may be the increased morbidity rate after highly invasive BTC surgery. The morbidity rate of the study was 40%, which seems favorable in comparison with the reported morbidity rate of 25.7%–57.3% [23, 24, 25, 26, 27]. One additional issue to consider is the risk of losing surgical opportunities due to disease progression during neoadjuvant chemotherapy. However, such cases typically have a very poor prognosis, and neoadjuvant chemotherapy may serve to identify cases where surgery would likely be futile due to early recurrence.

While our study was not designed to assess survival outcomes definitively, we observed encouraging trends in OS and PFS in BTC patients with FDG‐PET‐positive lymph nodes, which is highlighted considering the dismal survival data in previous studies. In this study, preoperative GCS chemotherapy offered a median OS of more than 30.0 months and median PFS of 17.3 months in BTC patients with FDG‐PET‐positive lymph nodes, in comparison with previous studies reporting a median OS of 5.6 months in extrahepatic BTC with FDG‐PET‐positive lymph nodes [7] and a mean DFS of 91 days in intrahepatic BTC with FDG‐PET‐positive lymph nodes [6]. Furthermore, among the 15 patients who underwent surgical resection, the 2‐year OS rate was 79.4%, which appears favorable compared to a previously reported 2‐year OS of 67% [30], despite the likelihood that the current cohort included more biologically aggressive BTC cases. These favorable survival outcomes of this study could be attributed to the high effectiveness of GCS chemotherapy. In unresectable BTC patients, the GCS regimen has exhibited promising efficacy, providing a high response rate (41.5%) and superior OS to GC chemotherapy (HR 0.79, 90% CI: 0.628–0.996; *p* = 0.046) [12]. In the neoadjuvant setting, the study demonstrated the radiological response rate was 12.5%. Among the FDG‐PET‐positive lymph nodes, FDG uptake disappeared in 23.8%, and pathological CR was confirmed in 27.8%. The discrepancy between the radiological response rate and the pathological CR rate in lymph nodes may be partially explained by the fact that tumor viability can decrease without substantial reduction in lesion size. These observations support further validation of neoadjuvant GCS chemotherapy in controlled trials, such as JCOG1920, which will assess its utility in broader patient populations beyond high‐risk patients with FDG‐PET‐positive lymph nodes.

Preoperative detection of lymph node metastasis is an issue of discussion. CT/MRI size alone has limited diagnostic accuracy [31, 32], with a reported positive predictive value of only 57% when using a short‐axis diameter ≥ 10 mm [33]. FDG‐PET CT has demonstrated a relatively low sensitivity of 57% for lymph node metastasis [15], suggesting a notable risk of false negatives. In contrast, the specificity of PET‐CT has been shown to be exceptionally high [15] and, when combined with the CT size criterion (≥ 10 mm), a positive predictive value of 100% has been reported [6, 7, 14, 15], in spite of the concern of false positives secondary to inflammatory changes. Thus, we adopted a combined criterion of CT size and FDG accumulation to ensure that enrolled patients truly had nodal metastasis.

Given the extremely poor prognosis associated with FDG‐PET‐positive lymph nodes in BTC, such cases can be considered borderline‐resectable or even oncologically unresectable despite being technically resectable. Similarly, limited para‐aortic lymph node metastases are also technically resectable but unsuitable for upfront surgery. Since preoperative chemotherapy is to suppress tumor progression and to select optimal surgical candidates among oncologically unsuitable for upfront resection, and the primary objective of this trial was to assess the safety and feasibility of neoadjuvant GCS chemotherapy in this high‐risk population, these two subgroups were treated as a single eligible population. This study concept partially overlaps with that of conversion surgery for initially unresectable disease [34], and the optimal classification of resectability in this context warrants further discussion.

This phase II study used GCS chemotherapy as a neoadjuvant chemotherapy regimen, which is one of the most effective chemotherapy regimens for patients with unresectable BTC [12]. Another neoadjuvant option is immunotherapy, based on recent phase III trials demonstrating the survival benefit of adding immunotherapy (e.g., durvalumab in TOPAZ‐1 [35] or pembrolizumab in KEYNOTE‐966 [36]) to standard chemotherapy (GC regimen) for unresectable BTC patients, although the higher response rate of 41.5% reported with GCS may offer an advantage in the neoadjuvant setting [12]. A phase II trial is also underway to compare neoadjuvant GC versus GC plus durvalumab in terms of R0 resection rate in resectable BTC (NCT04308174).

Our study has limitations, including a small sample size, relatively short follow‐up duration, and the absence of a control group. Some patients, especially those enrolled later in the trial, were censored at the time of analysis, which contributed to the observation that the median survival time exceeded the median follow‐up time. While we demonstrated the safety and feasibility of neoadjuvant GCS chemotherapy, our findings, including the potential survival benefits, need to be confirmed by the ongoing randomized phase III trial (JCOG1920) [20]. Second, the patient selection criteria focusing on FDG‐PET‐positive lymph nodes may introduce a form of selection bias, and potential variability in the sensitivity and specificity of PET imaging depending on the tumor location and characteristics may affect the generalizability of the study's findings. In four cases, FDG‐PET was not performed after GCS chemotherapy due to discontinuation of protocol chemotherapy/lack of insurance coverage, which could result in incomplete data and introduce bias into the study. Additionally, strictly distinguishing inflammation‐driven FDG uptake from tumor‐related FDG uptake remains challenging. However, we believe that by combining FDG‐PET with other imaging modalities and using clinical judgment, it is generally possible to differentiate between the two, mitigating this concern [33, 37]. Regarding the pathological CR rate of FDG‐PET‐positive lymph nodes, there is a potential bias leading to an overestimation of the CR rate due to the exclusion of cases that did not undergo lymph node retrieval and therefore were not included in the pathological examination. Although this study followed an intention‐to‐treat principle and included all enrolled patients in the analysis, the fact that some patients did not undergo surgery may introduce a degree of attrition bias. Finally, the inclusion of a single ethnic group may be another limitation in applying the findings of this study worldwide.

In conclusion, neoadjuvant GCS chemotherapy is a safe and feasible treatment option for patients with resectable BTC and FDP‐PET‐positive lymph node metastasis. The high completion rate and acceptable toxicity profile, along with the reasonable curative resection rate, support its use in this setting. Further large‐scale studies are warranted to evaluate its efficacy and potential survival benefits.

## Conflicts of Interest

The authors declare no conflicts of interest.

## Data Availability

Data supporting the findings of this study is available from the corresponding author upon reasonable request.
